# ﻿*Impatiensyunlingensis* (Balsaminaceae), a new species from Yunnan, China

**DOI:** 10.3897/phytokeys.212.89347

**Published:** 2022-10-27

**Authors:** Jiang-Hong Yu, Wen-Di Zhang, Fei Qin, Chang-Ying Xia, Ying Qin, Ming-Tai An, Sudhindra R. Gadagkar, Sheng-Xiang Yu

**Affiliations:** 1 College of Forestry, Guizhou University, Guiyang 550025, China; 2 State Key Laboratory of Systematic and Evolutionary Botany, Institute of Botany, Chinese Academy of Sciences, Beijing 100093, China; 3 University of Chinese Academy of Sciences, Beijing 100049, China; 4 Biomedical Sciences, College of Graduate Studies, Midwestern University, Arizona 85308, USA; 5 College of Veterinary Medicine, Midwestern University, Glendale, Arizona 85308, USA; 6 School of Life Sciences, Southwest University, Chongqing 400715, China; 7 Chongqing Key Laboratory of Plant Resource Conservation and Germplasm Innovation, Chongqing 400715, China; 8 Guangxi Institute of Botany, Guangxi Zhuang Autonomous Region and the Chinese Academy of Sciences, Guilin 541006, China; 9 College of Life Sciences, Guangxi Normal University, Guilin 541004, China

**Keywords:** Morphology, phylogeny, pollen grains, seed micromorphology, taxonomy

## Abstract

*Impatiensyunlingensis* S.X. Yu, Chang Y. Xia & J.H. Yu (Balsaminaceae), a species new to science discovered in Yunnan, China, is described and illustrated here, along with its phylogenetic position among other *Impatiens* species. Morphological, micro-morphological and molecular evidence is presented as an attestation of its novelty. *Impatiensyunlingensis* is similar to *I.delavayi* in having coarsely crenate leave margins, bracts in the upper part, ca. ^4^/_5_ length of the pedicels, saccate lower sepal with shallowly bifid spur, linear capsules, and elliptic-oblong, tuberculate seeds, but differs from *I.delavayi* with lateral sepals 4 (vs. 2), lateral united petal basal lobes subtriangular (vs. dolabriform), and seeds’ surface equipped with tubercular ornamentation mostly covered with grain shaped appendages (vs. glabrous and without grain shaped appendages on the top).

## ﻿Introduction

*Impatiens* L. (Balsaminaceae) is a large plant genus containing more than 1000 species, geographically distributed over a wide range, including tropical Africa, India, southwestern Asia, southern China, and Japan, with a few species having radiated into Europe, Siberia, northern China, and North America (Grey-Wilson 1980; Fischer 2004; Yu et al. 2016). The greatest amount of diversification in this genus is seen in tropical Africa, Madagascar, the Himalayas and mainland tropical Asia (Song et al. 2003; Yuan et al. 2004). Of these, the latter two, located in southwest China, account for more than 270 species of *Impatiens* (Chen 2001; Chen et al. 2007; Yu 2012).

The genus *Impatiens* was recognized as a “notoriously” difficult group for taxonomical purposes more than a century ago (Hooker 1908), and it has continued to retain that status (Grey-Wilson 1980; Chen 2001). This reputation is largely because of the prolific diversification in this genus, which is exacerbated by the paucity of well-preserved specimens because of the rather ephemeral nature of the fleshy and succulent stems and the extremely delicate sepals and petals. Still, new species are constantly being discovered in this remarkable genus. Southwest China is one of the regions that has seen a surge, with six species added from the provinces of Sichuan and Yunnan in recent years – *I.maculifera* S.X. Yu & Chang Y. Xia (Xia et al. 2019), *I.baishaensis* B. Ding & H.P. Deng (Ding et al. 2017), *I.wawuensis* Bo Ding & S.X. Yu (Ding et al. 2016), *I.pandurata* Y.H. Tan & S.X. Yu (Tan et al. 2015), *I.shimianensis* Ge Chen Zhang & L.B. Zhang (Luo et al. 2015), and *I.xanthinoides* G.W. Hu (Cai et al. 2015). Unfortunately, the habitats of these mostly endemic species are being destroyed, or at the very least, fragmented, by increased tourism and the associated developments in infrastructure, underscoring the need to urgently investigate the presence of other *Impatiens* species and their distribution in this part of the country.

Our lab has made a concerted effort toward this end over the past few years by means of several expeditions into various regions of Southwest China, during which we discovered several species new to science of *Impatiens* (Bi et al. 2009; Yu et al. 2013; Tan et al. 2015; Ding et al. 2016; Xia et al. 2019). We now believe, based on a specimen collected from Northwest Yunnan in 2018, that we have found yet another species of *Impatiens* that is new to science. In the following, we describe its unique gross morphology and the micro-morphology of the pollen grains and seed coat, and discuss its relationships with its most closely related species (*I.delavayi* Franch., as determined by its phylogenetic position).

## ﻿Materials and methods

### ﻿Morphology

Morphological characters, such as leaves, inflorescences, flowers, and capsules, were carefully observed and measured in the field, followed by description and illustration in the lab, with meticulous attention to detail. In addition, we compared the specimen with related species based on field notes and photographs taken during the expedition, as well as with FAA-fixed material and dried specimens from PE (abbreviation follows Thiers 2022).

### ﻿Pollen grains and seeds

Mature, whole pollen grains and seeds collected from the field were observed directly and measured under magnification using an anatomical lens. Subsequently, they were mounted on double-sided adhesive tape and coated with a layer of gold before being photographed using a Hitachi S-4800 SEM. The micro-morphological characters were described following Wang and Wang (1983) and Lu (1991) for pollen grains, and Lu and Chen (1991), Liu et al. (2004), and Song et al. (2005) for seeds. The average size of pollen grains and seeds was calculated based on 20 of each.

### ﻿Taxon sampling

We used a total of 152 species of *Impatiens* in this study, including three individuals of the putative new species, and three outgroup species: *Hydroceratriflora* (L.) Wight & Arn. (Balsaminaceae), *Marcgraviaumbellata* L., and *Noranteaguianensis* Aubl. (Marcgraviaceae) that were, included following Yuan et al. (2004), Janssens et al. (2006), and Yu et al. (2016). We downloaded DNA sequences for two molecular markers (see below) for all the species used, from GenBank except for the specimen under consideration, for which, they were newly generated for this study. Species names and GenBank accession numbers are listed in Suppl. material 1: Table S1.

### ﻿DNA extraction, PCR amplification, and sequencing

We used two molecular markers in the study: ITS (ITS-1, 5.8S, and ITS-2) and *atp*B-*rbc*L. For the putative new species, we extracted total genomic DNA from silica gel-dried leaves using a CTAB protocol modified from that of Doyle and Doyle (1987). For the primers and PCR protocols for ITS and *atp*B-*rbc*L, we followed White et al. (1990) and Taberlet et al. (1991), respectively. Subsequently, we purified the PCR products using a GFX^TM^PCR DNA and Gel Band Purification Kit (Amersham Pharmacia Biotech, Piscataway, NJ, USA), and sequenced the markers using an ABI Prism Bigdye Terminator Cycle Sequencing Kit (Applied Biosystems, Foster City, CA, USA), while analyzing the PCR products on an ABI3730xl automated DNA sequencer.

### ﻿Phylogenetic analysis

Sequences were aligned using the default parameters in Clustal X v.1.83 (Thompson et al. 1997) and subsequently adjusted manually in BioEdit v.7.0 (Hall 1999). One difficult-to-align region in *atp*B-*rbc*L (encompassing 42 sites) was excluded from the analyses. Bayesian inference (BI) was used to analyze the ITS and plastid data sets, by means of MrBayes v.3.0b4 (Ronquist and Huelsenbeck 2003). Both regions (ITS and *atp*B-*rbc*L) were assigned the GTR+I+G model of nucleotide substitution, as determined by the Akaike information criterion (AIC) in Modeltest v.3.06 (Posada and Crandall 1998).

## ﻿Taxonomic treatment

### ﻿ Impatiens yunlingensis

Taxon classificationPlantaeEricalesBalsaminaceae

S.X. Yu, Chang Y. Xia & J.H. Yu sp. nov.

46E762A9-6C97-5D8D-8D69-42A4D8EF6DCD

urn:lsid:ipni.org:names:77307296-1

Figs 1 , 2

#### Diagnosis.

Similar to *I.delavayi* Franchet, in having coarsely crenate leave margin, bracts in the upper part, ca. 4/5 length of the pedicels, saccate lower sepal with shallowly bifid spur, but differs from *I.delavayi* with lateral sepals 4 (vs. 2) and lateral united petal basal lobes subtriangular (vs. dolabriform).

#### Type.

China. Yunnan: Dêqên County, Yunling Township, Yongzhi Village, understory and along river, alt. 1780 m, 28°11'N, 98°49'E, 07 Oct. 2018, Shengxiang Yu, Changying Xia, Xuexue Wu and Xiaxing Liu 9998 (holotype: PE, isotype: IBK).

**Figure 1.**
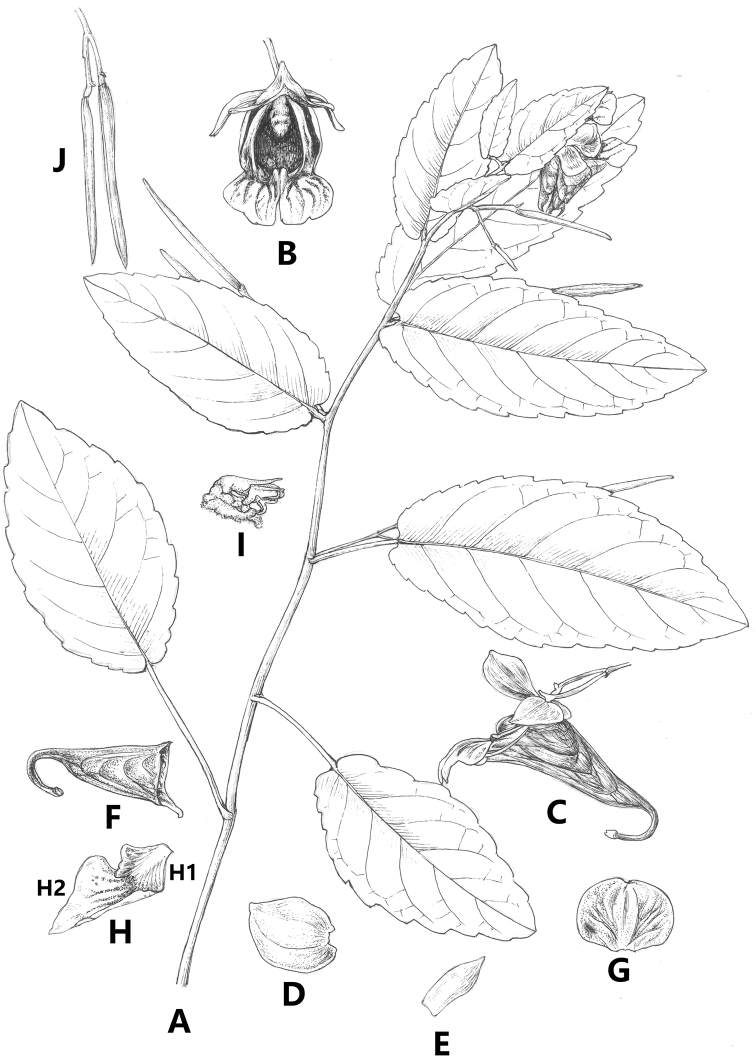
*Impatiensyunlingensis* S.X. Yu, Chang Y. Xia & J.H. Yu, sp. nov. **A** branch with leaves, flowers and capsules **B** flower, front view **C** flower, lateral view **D** outer lateral sepal **E** inner lateral sepal **F** lower sepal **G** dorsal petal **H** lateral united petals (H1) basal lobe (H2) distal lobe **I** filaments and anthers **J** capsules (Drawing by Wen-Hong Lin).

#### Description.

Annual herb, 50–70 cm tall. Stems slender, erect, branched, or simple, glabrous; inferior nodes swollen, glabrous. Leaves alternate; lower and middle leaves petiolate, upper leaves sessile or subsessile; petiole 2–3 cm long, slender, purplish or pale green, glabrous, glandless; leave blade, 5–8 cm long, 3–5 cm wide, broadly ovate or ovate-orbicular, base cordate, apex obtuse, margin coarsely crenate, thinly membranous, glabrous; lateral vein 5–7 pairs; margin coarsely crenate. Lower and middle leaves petiolate; petiole 2–3 cm long, glabrous, purplish, slender; Racemes in upper axils, 2–3-flowered; peduncles 2–4 cm long, slender. Pedicels 1–3 cm long, glabrous, purplish, bracteate below flowers; bracts ovate, in the upper part, ca. ^4^/_5_ length of the pedicels, ca. 1 mm wide, 1–3 mm long, acute, persistent. Flowers purplish, large, 2.5–3.5 cm deep. Lateral sepals 4; outer 2 large, 1–1.5 cm long, ca. 1 cm wide, 1–3 mm long, obliquely ovate, inequilateral, apex acute, glabrous, purplish; inner 2 small, 2–4 mm long, 1–1.5 mm wide, oblong, apex acuminate glabrous, purplish or pale green. Lower sepal 2–2.5 cm deep, 1.5–2 mm wide, 2.5–3 cm long, saccate, purplish red, abruptly narrowed into an incurved spur; spur short, ca. 1 cm long, shallowly bifid. Dorsal petal 8–12 mm long, 10–15 mm wide, orbiculate, base truncate, apex rounded, glabrous, purple, midrib thickened. Lateral united petals 2.5–3 cm long, 2-lobed; basal lobes ca. 1 cm long, 5 mm wide, subtriangular, apex obtuse, glabrous, purplish; distal lobes 2.5–3 cm long, 8–12 mm wide, triangular, apex acute glabrous, purplish or buff; auricle inflexed. Stamens 5, anthers obtuse. Capsule linear, 3–4 cm long, five carpels, many seeds per locule. Seeds elliptic-oblong, tuberculate.

**Figure 2.**
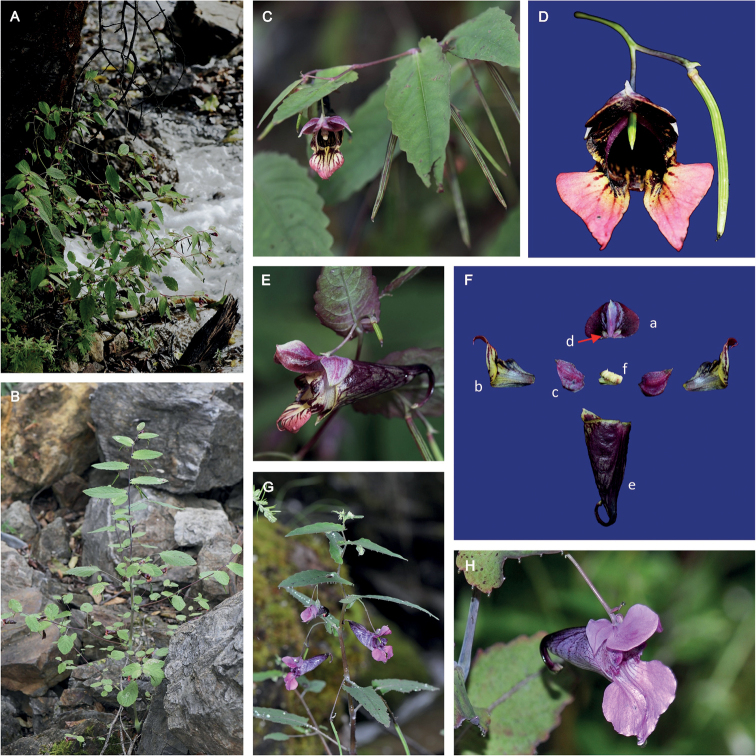
**A–F***Impatiensyunlingensis***A** habitat **B** plant **C** branch with flower **D** flower, front view **E** flower, lateral view **F** flower anatomy (a) dorsal petal (b) lateral united petals (c) outer lateral sepal (d) inner lateral sepal (e) lower sepal (f) filaments and anthers **G***Impatiensdelavayi* branch with flowers **H***Impatiensdelavayi* flower.

#### Phenology.

Flowering occurs from September to October, fruiting from September to November.

#### Distribution and ecology.

**Figure 3.**
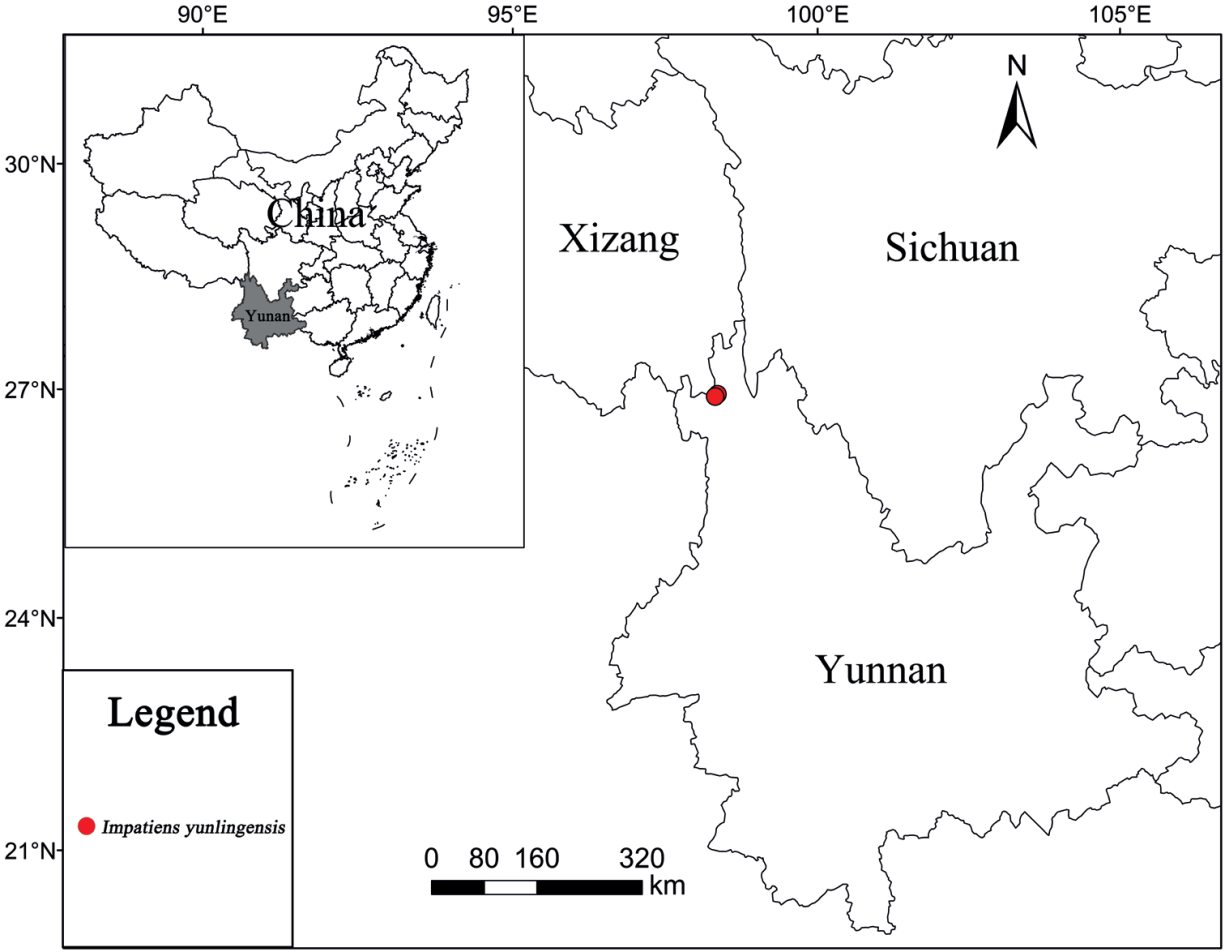
Geographical distribution of *Impatiensyunlingensis*.

This species is only known to be found in Dêqên County, Yunnan, China (Fig. 3); under evergreen broadleaf forests and along the river; alt. 1780–2500 m.

#### Conservation status.

*Impatiensyunlingensis* is only known from the type locality with two middle-sized populations in an area that has been severely disturbed by overgrazing. Based on lack of additional local studies, we consider its status as Data Deficient [DD] (IUCN 2017). However, since the Hengduan Mountains are characterized by the high diversity of narrow-ranged species, including endemic, threatened, rare, and nationally protected species (Qin et al. 2017; Sun et al. 2017; Xu et al. 2019), all of which have been considered as a conservation priority for its global biodiversity hotspot and high species richness (Myers et al. 2000; Brooks et al. 2006; Xu et al. 2019). The conservation status of this apparently rare and narrow-ranged species is of high concern.

#### Etymology.

The specific epithet ‘*yunlingensis*’ refers to the locality of the type specimen, Yunling Township, Dêqên County, Yunnan, China.

#### Additional specimen examined.

***Paratype*.** China. Yunnan: Dêqên County, Yunling Township, Shualao Village; hillside and understory, alt. 2500 m, 28°09'N, 98°47'E, 07 Oct. 2018, Shengxiang Yu, Changying Xia, Xuexue Wu and Xiaxing Liu 10002 (PE).

#### Seed description and palynology.

**Figure 4.**
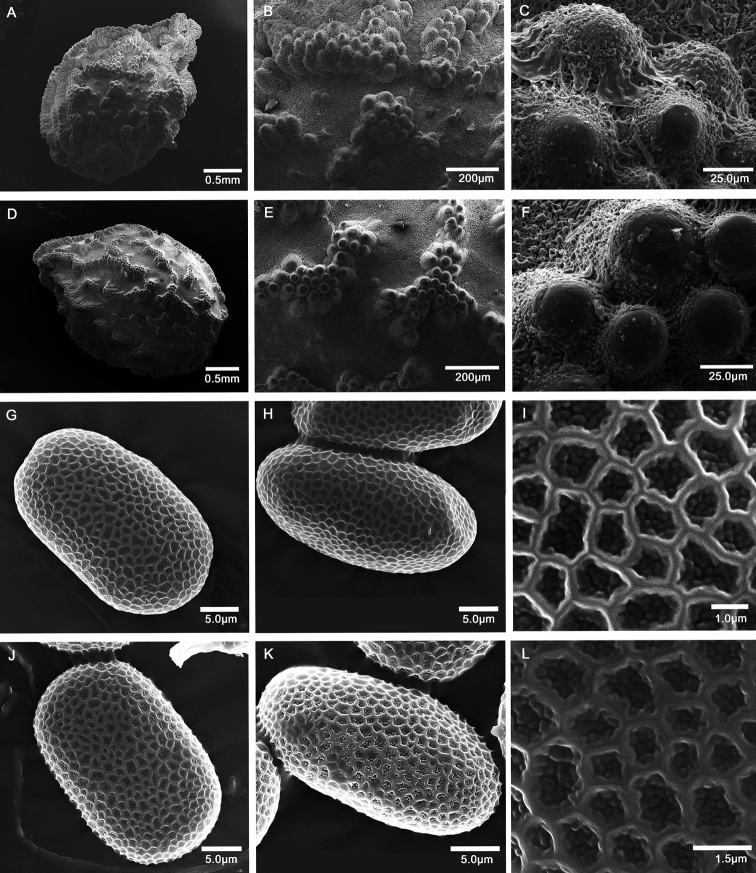
Scanning electron microscope images of seeds and pollen grains **A–C** seeds of *Impatiensyunlingensis***D–F** seeds of *I.delavayi***G–I** pollen grains of *I.yunlingensis***J–L** pollen grains of *I.delavayi*.

Seeds of *I.yunlingensis* are elliptic-oblong, with a size of 2.6 × 1.9 mm, L (long) / W (wide) = 1.37 (Fig. 4 A–C). The surface is equipped with coarse tubercular ornamentation mostly covered by grain-shaped appendages. While the seeds of *I.delavayi* are also elliptic-oblong, with a size of 3.5 × 2.3 mm, L (long) / W (wide) = 1.52 (Fig. 4 D–F), the surface contains coarse tubercular ornamentation glabrous and no grain shaped appendages on the top. Pollen grains of both *I.yunlingensis* and *I.delavayi* are tetracolpate, elliptic in polar view, exine with irregular reticulate ornamentation, and granules in lumina. *Impatiensyunlingensis* pollen size (E_1_ × E_2_: length of long equatorial axis × length of short equatorial axis) is 28.05 × 16.67 μm (Fig. 4 G–I), while it is 27.78 × 17.50 μm (E_1_ × E_2_) for *I.delavayi* (Fig. 4J–L) (Suppl. material 1: Table S2).

## ﻿Results

### ﻿Nuclear data phylogenetic analyses

**Figure 5.**
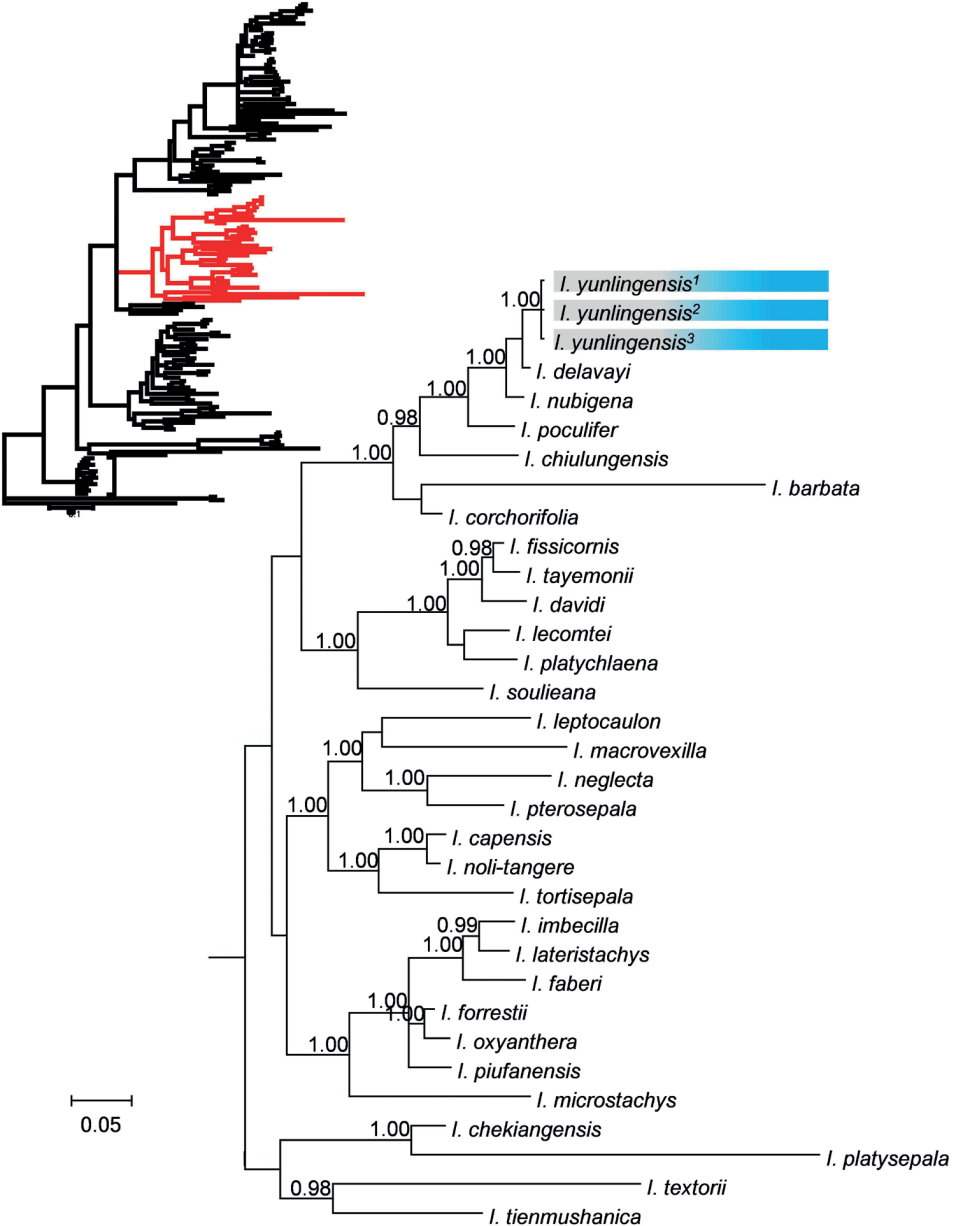
Partial Bayesian consensus phylogram based on ITS sequences. Numbers above branches are Bayesian posterior probabilities (only PP values > 0.95 shown).

**Figure 6.**
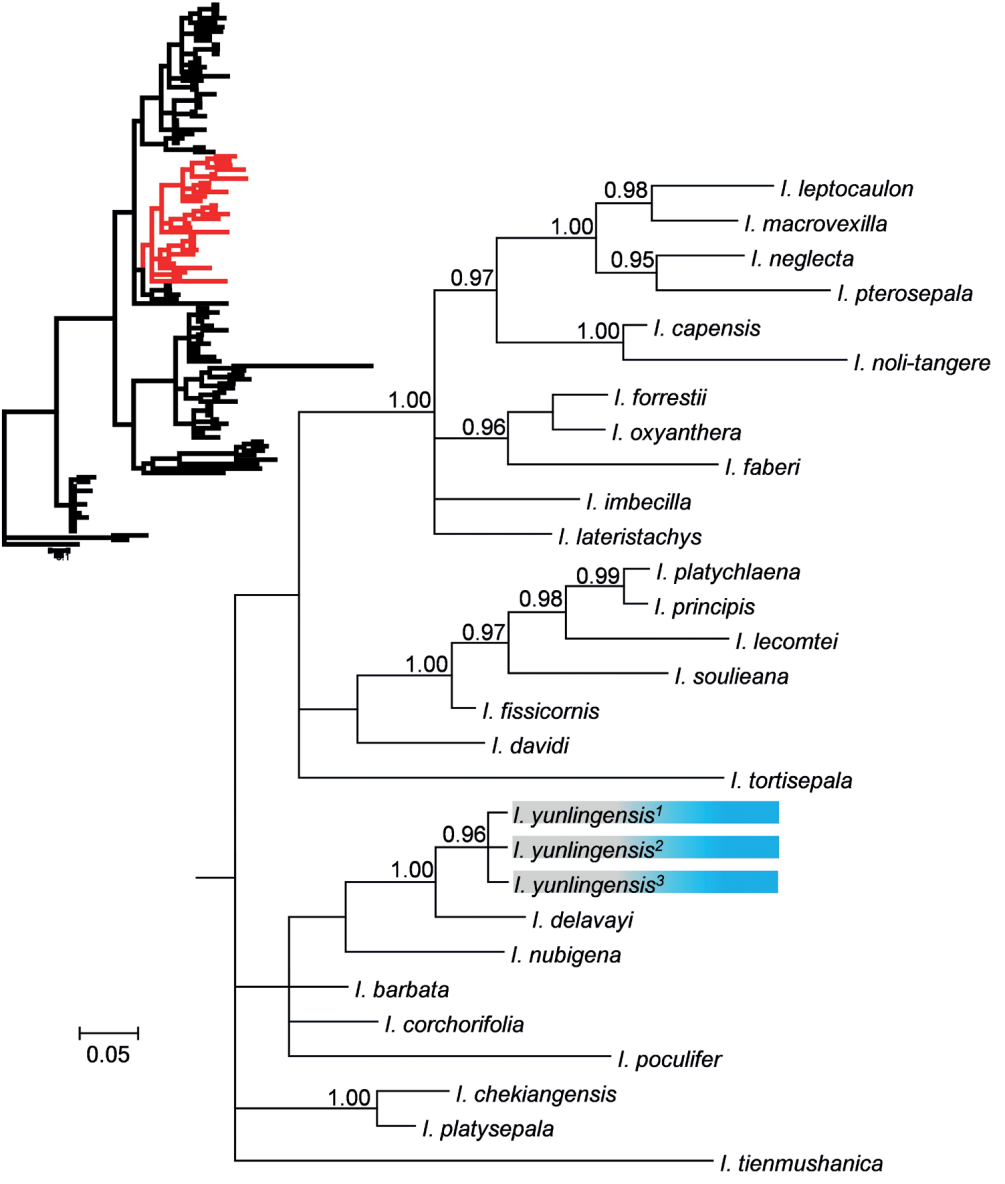
Partial Bayesian consensus phylogram based on *atp*B-*rbc*L sequences. Numbers above branches are Bayesian posterior probabilities (only PP values > 0.95 shown).

Although phylogenetic analysis was done using all 152 species, we only show a few clades here, along with the position of the root (Figs 5 and 6) (see Suppl. material 1: Figs S1 and S2 for the tree with all the species). Fig. 5 shows that the three individuals of *I.yunlingensis* cluster together in the phylogenetic tree of ITS with strong support (PP = 1.00), and the tree shows that *I.yunlingensis* is the sister species of *I.delavayi*, although with relatively poor support (PP < 0.95). *Impatiensnubigena* W.W. Smith has the closest relationship to *I.yunlingensis* and *I.delavayi* (PP = 1.00), followed by *I.poculifer* Hook. f. (PP = 1.00), and *I.chiulungensis* Y.L. Chen (PP = 0.98).

### ﻿Plastid data phylogenetic analysis

The three individuals of *I.yunlingensis* clustered together with strong support (PP = 0.96) in the phylogenetic tree of *atp*B-*rbc*L also (Fig. 6), with *I.delavayi* once again as the sister species, and with strong support (PP = 1.00). As in the ITS tree (Fig. 5), *I.nubigena* has the closest relationship to *I.yunlingensis* and *I.delavayi*, but with poor support, and the clade of these three species is in a polytomy with *I.barbata* Comber, *I.corchorifolia* Franch., and *I.poculifer*.

While the ITS tree (Fig. 5) has a higher resolution and more numbers of internal nodes with high support, both tree topologies resemble each other and are also similar to those obtained in previous studies (Yuan et al. 2004; Janssens et al. 2006; Yu et al. 2016; Ruchisansakun et al. 2021).

Based on the position of *I.yunlingensis* in both trees, we conclude that it is a new species to science that belongs to the subgenus Impatiens (Yu et al. 2016). This evidence is corroborated by the morphological features of *I.yunlingensis* as well, which are in accordance with those of subg. Impatiens, e.g., 2-flowered inﬂorescences and linear fruits (Yu et al. 2016).

## ﻿Discussion

Both phylogenetic trees (ITS and *atp*B-*rbc*L; Figs 5 and 6, respectively) indicate that *I.yunlingensis* is a distinct member of the genus, and furthermore, support its sister taxon relationship with *I.delavayi*, thus corroborating the evidence provided by the morphological and micro-morphological observations. There are two populations of *I.yunlingensis* that have been recorded and observed, and we find that the morphological characters of the species present consistency between the two populations, especially with respect to the morphology and number of lateral sepals (4 lateral sepals including the outer 2 and inner 2). *Impatiensyunlingensis* is similar to *I.delavayi* in having coarsely crenate leaf margins, bracts in the upper part, ca. ^4^/_5_ length of the pedicels, saccate lower sepal with shallowly bifid spur, linear capsules, and elliptic-oblong, tuberculate seeds, but differs from *I.delavayi*, with lateral sepals 4 (vs. 2), lateral united petal basal lobes subtriangular (vs. dolabriform), and seeds surface contain tubercular ornamentation mostly covered by grain shaped appendages (vs. glabrous and without grain shaped appendence on the top). This feature of lateral sepals 4 is crucial for distinguishing *I.yunlingensis* from *I.delavayi* and other related species, and supports its standing as a separate, and new species. It is worth noting that the morphological characters of *I.delavayi* are consistent in its distribution range. We examined all the specimens of *I.delavayi* preserved in PE and found that there were only two lateral outer sepals, with no lateral inner sepals, not even rudimentary ones. Furthermore, to our knowledge, there is no report of variation in the lateral sepal number of *I.delavayi*. Therefore, we believe that the number of lateral sepals is a reliable trait for this purpose.

As pointed out by previous studies, the characteristic of 4 sepals is seen in many species of section Semeiocardium (Ruchisansakun et al. 2015) and in *I.oblongata* (sect. Impatiens) (Ruchisansakun et al. 2018). Indeed, only a few species of subgenus Impatiens exhibit this character, such as *I.barbata*, *I.chiulungensis*, and *I.chochorifolia*. However, other morphological features of these species clearly distinguish them from *I.yunlingensis*. For example, *I.barbata* is characterized by yellow-haired flowers and the plant is puberulent, and the latter two species can be readily differentiated from *I.yunlingensis* by the apex of the basal lobe, and the distal lobe of lateral united petal narrowing into a single long and hair-like appendage, respectively.

Our phylogenetic analyses generated a result consistent with previous studies (Yuan et al. 2004; Janssens et al. 2006; Yu et al. 2016; Ruchisansakun et al. 2021), indicating that the *I.yunlingensis* belongs to subg.Impatiens. The ITS-based phylogenetic tree has a higher resolution and contains more nodes with high support, but it does not provide sufficient support for the relationship between *I.yunlingensis* and its close relatives, while the *atp*B-*rbc*L tree does. It is also worth noting that while the position of the clade itself is different between the two trees, at least two of the three previously mentioned species with the character of lateral sepals 4 (*I.barbata*, *I.chiulungensis*, and *I.chochorifolia*) are nested in the same large clade as *I.yunlingensis* in both phylogenetic trees. In summary, *I.yunlingensis*, with its morphological, micro-morphological, and phylogenetic distinctiveness, adds another external node to the growing *Impatiens* phylogeny and should help in elucidating the evolutionary significance of the genus, particularly with respect to its propensity for diversification.

**Additional specimen examined.***Impatiensdelavayi* Franch—China. Sichuan: Kangwu Temple, near Muli Bridge, Muli County, Sichuan Province, 21 Feb 2012, *S.X. Yu, Y.T. Hou, X.X. Zhang & Y.M. Zhao* 4664 (PE). Xizang: Zayü County, Xizang, alt. 3700 m, 27 Sep 1982, *Qinghai-Tibet expedition* 10807 (PE); Dzer-nar, Tsa-wa-rung, Xizang, alt. 3000 m, Sep 1935, *C.W. Wang* 66212 (PE). Yunnan: East slope of Haba Snow Mountain, Zhongdian County, Yunnan Province, alt. 3500–3800 m, 11 Aug 1981, *Hengduan Mountains Research Team, Institute of Botany, the Chinese Academy of Sciences* 2938 (PE); Yulong Mountains, Lijiang County, Yunnan Province, alt. 3200 m, 6 Aug. 1959, *anonymous* 22522 (PE); Zhongdian County, Yunnan Province, 27°27'33"N, 99°55'2"E, alt. 3050 m, 26 July 2006, *D.E. Boufford, S.L. Kelley, R.H. Ree, H. Sun, B. Xü, J.P. Yue, D.C. Zhang & W.D. Zhu* 35372 (PE); Wei-se County, Yunnan Province, alt. 2600 m, 15 Sep 1934, *anonymous* 57925 (PE); Huan-fu-ping, A-tun-tze, Dêqên County, Yunnan Province, alt. 3500 m, Aug 1935, *C.W. Wang* 69058 (PE).

## Supplementary Material

XML Treatment for Impatiens yunlingensis

## Supplementary materials

Supplementary material 1 Appendix S1 Data type Morphological, phylogenetic, GenBank accession numbers, images. Explanation note **Table S1.** Species and GenBank accession numbers for the marker sequences used in this study **Table S2.** Morphological characteristics of *Impatiensyunlingensis* and *I.delavayi***Figures S1, S2.** Bayesian posterior probabilities figure. https://binary.pensoft.net/file/762598This dataset is made available under the Open Database License (http://opendatacommons.org/licenses/odbl/1.0/). The Open Database License (ODbL) is a license agreement intended to allow users to freely share, modify, and use this Dataset while maintaining this same freedom for others, provided that the original source and author(s) are credited.Jiang-Hong Yu, Ming-Tai An, Fei Qin, Chang-Ying Xia, Ying Qin, Sudhindra R. Gadagkar, Sheng-Xiang Yu
