# Supplementary material for: ﻿Impatiensyunlingensis (Balsaminaceae), a new species from Yunnan, China
Source: PhytoKeys. 2022 Oct 27;212:13–27. doi: 10.3897/phytokeys.212.89347 (PMC9836640; doi:10.3897/phytokeys.212.89347)
Supplement: Supplementary material 1 — Appendix S1 [file phytokeys-212-013_article-89347__-s001.doc]

**Supporting Information**

**Table S1. Species and GenBank accession numbers for the marker sequences used in this study.**

| Species | GenBank Accession number | | |
| --- | --- | --- | --- |
|  | ITS | *atpB-rbcL* | |
| *Impatiens acehensis* Grey-Wilson | AY348739 |  | |
| *I. amoena* H. Perrier | AY348795 |  | |
| *I. amphorata* Edgew. | AY348740 |  | |
| *I. andohahelae* Eb. Fisch. & Rahelivololona | AY348741 |  | |
| *I. andringitrensis* H. Perrier | AY348742 |  | |
| *I. angulata* S.X. Yu, Y.L. Chen & H.N. Qin | KP776060 |  | |
| *I. anovensis* H. Perrier | AY348743 |  |  |
| *I. apalophylla* Hook. f. | KP776061 | KP776011 |  |
| *I. aquatilis* Hook. f. | AY348745 | DQ147811 |  |
| *I. arguta* Hook. f. & Thomson | AY348746 | DQ147812 |  |
| *I. aureliana* Hook. f. | AY348747 | DQ147814 |  |
| *I. auricoma* Baill. | AY348748 | DQ147815 |  |
| *I. balansae* Hook. f. | KP776062 | KP776012 |  |
| *I. balsamina* L. | AY348749 | DQ147816 |  |
| *I. barbata* H. F. Comber | AY348750 | DQ147818 |  |
| *I. baronii* Baker | AY348751 |  |  |
| *I. begoniifolia* S. Akiyama & H. Ohba | AY348752 | DQ147819 |  |
| *I. bequaertii* De Wild. | AY348753 | DQ147820 |  |
| *I. bicornuta* Wall. | AY348754 | DQ147821 |  |
| *I. blinii* Lévl. | KP776063 | KP776013 |  |
| *I. bombycina* W. Lobin & E. Fischer | AY348755 |  |  |
| *I. brachycentra* Kar. & Kir. | AY348756 |  |  |
| *I. burtonii* Hook. f. | AY348757 |  |  |
| *I. campanulata* Wight | AY348758 | DQ147822 |  |
| *I. capensis* Meerb. | AY348759 | DQ147823 |  |
| *I. chekiangensis* Y.L. Chen | KP776064 | KP776014 |  |
| *I. chinensis* L. | AY348761 | DQ147825 |  |
| *I. chishuiensis* Y.X. Xiong | KP776065 | KP776015 |  |
| *I. chiulungensis* Y.L. Chen | KP776066 | KP776016 |  |
| *I. chlorosepala* Hand. -Mazz. | KP776067 | KP776017 |  |
| *I. claeri* N. Halle | AY348763 |  |  |
| *I. clavigera* Hook. f. | KP776068 | KP776018 |  |
| *I. auriculata* (S. H. Huang) S. X. Yu | MN095283 | MN095284 |  |
| *I. columbaria* J. J. Bos | AY348764 | DQ147828 |  |
| *I. conchibracteata* Y.L. Chen | AY348765 | DQ147829 |  |
| *I. congolensis* G. M. Schulze & R. Wilczek | AY348766 | DQ147830 |  |
| *I. corchorifolia* Franch. | AY348767 | DQ147831 |  |
| *I. cordata* Wight | AY348768 |  |  |
| *I. cuspidata* Wight & Arn. | AY348769 | DQ147832 |  |
| *I. cyanantha* Hook. f. | AY348770 | DQ147833 |  |
| *I. cyathiflora* Hook. f. | AY348771 | DQ147834 |  |
| *I. cymbifera* Hook. f. | KP776069 | KP776019 |  |
| *I. davidi* Franch. | KP776070 | KP776020 |  |
| *I. delavayi* Franch. | AY348773 | DQ147836 |  |
| *I. desmantha* Hook. f. | AY348774 | DQ147837 |  |
| *I. drepanophora* Hook. f. | AY348776 | DQ147838 |  |
| *I. duclouxii* Hook. f. | KP776071 | KP776021 |  |
| *I. eubotrya* Miq. | AY348777 |  |  |
| *I. faberi* Hook. f. | AY348778 | DQ147841 |  |
| *I. falcifer* Hook. f. | KP776072 | KP776022 |  |
| *I. firmula* Baker | AY348780 |  |  |
| *I. fischeri* Warb. | AY348781 | DQ147843 |  |
| *I. fissicornis* Maxim. | AY348782 | DQ147844 |  |
| *I. flanaganae* Hemsl | AY348783 | DQ147846 |  |
| *I. forrestii* Hook. f. ex W. W. Smith | AY348784 | DQ147847 |  |
| *I. fragicolor* C. Marquand & Airy Shaw | KP776073 | KP776023 |  |
| *I. fuchsioides* H. Perrier | AY348785 |  |  |
| *I. furcata* H. Perrier | AY348786 |  |  |
| *I. gibbosa* H. Perrier | AY348787 |  |  |
| *I. gongshanensis* Y.L. Chen | KP776074 | KP776024 |  |
| *I. harae* H. Ohba & S. Akiyama | KP776075 | KP776025 |  |
| *I. henslowiana* Arn. | AY348790 |  |  |
| *I. hians* Hook. f. | AY348791 | DQ147849 |  |
| *I. hoehnelii* T. C. E. Fr. | AY348792 |  |  |
| *I. holocentra* Hand. -Mazz. | AY348793 |  |  |
| *I. hongkongensis* Grey-Wilson | KP776076 | KP776027 |  |
| *I. hunanensis* Y.L. Chen | KP776077 | KP776028 |  |
| *I. imbecilla* Hook. f. | AY348796 | DQ147851 |  |
| *I. inaperta* H. Perrier | AY348797 | DQ147852 |  |
| *I. keilii* Gilg. | AY348798 | KP776029 |  |
| *I. kerriae* Craib | AY348799 | DQ147853 |  |
| *I. kilimanjari* Oliver | AY348800 |  |  |
| *I. lateristachys* Y.L. Chen & Y. Q. Lu | KP776078 | KP776030 |  |
| *I. laxiflora* Edgew | KP776079 | KP776031 |  |
| *I. lecomtei* Hook. f. | AY348802 | DQ147855 |  |
| *I. leptocaulon* Hook. f. | KP776080 | KP776032 |  |
| *I. leschenaultii* Wall. | AY348803 | DQ147856 |  |
| *I. levingei* Gamble ex Hook. f. | AY348804 |  |  |
| *I. loburifera* S.X. Yu | KP776081 | KP776033 |  |
| *I. macrovexilla* Y.L. Chen | KP776082 | KP776034 |  |
| *I. manaharensis* Baill. | AY348805 |  |  |
| *I. margaritifera* Hook. f. | KP776084 | KP776036 |  |
| *I. mengtzeana* Hook. f. | AY348806 | DQ147858 |  |
| *I. meruensis* Gilg. | AY348807 | DQ147859 |  |
| *I. microstachys* Hook. f. | KP776085 |  |  |
| *I. miniata* Grey-Wilson | AY348809 |  |  |
| *I. monticola* Hook. f. | AY348810 | DQ147860 |  |
| *I. morsei* Hook. f. | KP776086 | KP776037 |  |
| *I. napoensis* Y.L. Chen | AY348811 | DQ147861 |  |
| *I. neglecta* Y.L. Xu & Y.L. Chen | KP776087 | KP776038 |  |
| *I. niamniamensis* Gilg. | AY348812 | DQ147862 |  |
| *I. noli-tangere* L. | KP776088 | KP776039 |  |
| *I. nubigena* W. W. Smith | KP776089 | KP776040 |  |
| *I. nyimana* C. Marquand & Airy-Shaw | KP776090 | KP776041 |  |
| *I. obesa* Hook. f. | KP776091 | KP776042 |  |
| *I. omeiana* Hook. f. | KP776092 | DQ147864 |  |
| *I. oxyanthera* Hook. f. | AY348814 | DQ147865 |  |
| *I. pandurata* Y. H. Tan & S. X. Yu | KU042074 | KU042072 |  |
| *I. parasitica* Bedd. | AY348815 |  |  |
| *I. parviflora* DC. | AY348816 | DQ147866 |  |
| *I. percrenata* H. Perrier | AY348817 |  |  |
| *I. piufanensis* Hook. f. | KP776094 |  |  |
| *I. pingxiangensis* H.Y. Bi & S.X. Yu | KP776093 | KP776043 |  |
| *I. platychlaena* Hook. f. | AY348818 | DQ147867 |  |
| *I. platypetala* Lindl. | AY348819 | DQ147868 |  |
| *I. platysepala* Y.L. Chen | KP776095 | KP776044 |  |
| *I. poculifer* Hook. f. | AY348820 | DQ147870 |  |
| *I. principis* Hook. f. | KP776096 | KP776026 |  |
| *I. pritzelii* Hook. f. | AY348821 | KP776045 |  |
| *I. pseudoviola* Gilg. | AY348822 | DQ147871 |  |
| *I. pterosepala* Hook. f. | KP776097 | KP776046 |  |
| *I. purpurea* Hand.-Mazz. | AY348823 | DQ147872 |  |
| *I. racemosa* DC. | KP776098 | DQ147873 |  |
| *I. radiata* Hook. f. | AY348824 | KP776047 |  |
| *I. rectangula* Hand.-Mazz. | AY348825 | DQ147874 |  |
| *I. rothii* Hook. f. | AY348827 |  |  |
| *I. rubrostriata* Hook. f. | AY348828 | DQ147876 |  |
| *I. sambiranensis* H. Perrier | AY348829 |  |  |
| *I. scabrida* DC. | KP776099 | DQ147877 |  |
| *I. scullyi* Hook. F. | KP776100 | KP776048 |  |
| *I. scutisepala* Hook. f. | AY348830 | DQ147878 |  |
| *I. siculifer* Hook. f. | KP776101 | KP776049 |  |
| *I. sodenii* Engl. & Warb. ex Engl. | AY348832 | DQ147879 |  |
| *I. soulieana* Hook. f. | AY348833 | DQ147880 |  |
| *I. spathulata* Y.X. Xiong | KP776102 | KP776050 |  |
| *I. stenosepala* Pritz. ex Diels | AY348835 | DQ147881 |  |
| *I. stuhlmannii* Warb. | AY348836 |  |  |
| *I. subabortiva* H. Perrier | AY348837 |  |  |
| *I. sulcata* Wall. | KP776103 | KP776051 |  |
| *I. sunkoshiensis* S. Akiyama, H. Ohba & Wakab. | KP776104 | KP776052 |  |
| *I. taronensis* Hand. -Mazz. | AY348838 | DQ147882 |  |
| *I. tayemonii* Hayata | AY348839 |  |  |
| *I. teitensis* Grey-Wilson | AY348840 | DQ147883 |  |
| *I. textorii* Miquel | AY348841 |  |  |
| *I. tienmushanica* Y.L. Chen | KP776105 | KP776053 |  |
| *I. tinctoria* A. Rich. |  | FJ826677 |  |
| *I. tortisepala* Hook. f. | KP776106 | KP776054 |  |
| *I. trichosepala* Y.L. Chen | AY348843 | DQ147885 |  |
| *I. tuberculata* Hook. f. & Thomson | KP776107 | KP776055 |  |
| *I. tuberosa* H. Perrier | AY348844 | DQ147886 |  |
| *I. tubulosa* Hemsl. ex F. B. Forbes & Hemsl. | KP776108 | KP776056 |  |
| *I. uliginosa* Franch. | AY348845 | DQ147887 |  |
| *I. urticifolia* Wall. | KP776109 | AB043527 |  |
| *I. usambarensis* Grey-Wilson | AY348847 | DQ147890 |  |
| *I. vilersi* Costantin & Poisson | AY348848 |  |  |
| *I. walleriana* Hook. f. | AY348849 | DQ147892 |  |
| *I. wenshanensis* S.H. Huang | KP776110 | KP776057 |  |
| *I. wilsonii* Hook. f. | KP776111 | KP776058 |  |
| *I. xanthina* H. F. Comber | AY348850 | DQ147893 |  |
| *I. yaoshanensis* K.M. Liu & Y.Y. Cong | KP776112 | KP776059 |  |
| *I. yunlingensis* S.X. Yu, Chang Y. Xia & J.H. Yu^1^ | XXXXXXXX | XXXXXXXX |  |
| *I. yunlingensis* S.X. Yu, Chang Y. Xia & J.H. Yu ^2^ | XXXXXXXX | XXXXXXXX |  |
| *I. yunlingensis* S.X. Yu, Chang Y. Xia & J.H. Yu ^3^ | XXXXXXXX | XXXXXXXX |  |
| *I. zenkeri* (Warb.) Grey-Wilson | AY348852 |  |  |
| *Hydrocera triflora* (L.) Wight & Arn. | AY348853 | DQ147895 |  |
| *Marcgravia umbellata* L. |  | DQ147897 |  |
| *Norantea guianensis* Aubl. | AY348855 | DQ147898 |  |

**Table S2.** Morphological characteristics of *Impatiens yunlingensis* and *I. delavayi*

| Characters | *Impatiens yunlingensis* | *Impatiens delavayi* |
| --- | --- | --- |
| Seed shape | elliptic oblong | elliptic oblong |
| Seed size | a size of 2.6 × 1.9 mm, L (long) / W (wide) = 1.37 | a size of 3.5 × 2.3 mm, L (long) / W (wide) = 1.52 |
| Seedcoat | coarse tubercular ornamentation mostly covered by grain shaped appendages | contains coarse tubercular ornamentation with no grain shaped appendages on the top |
| Palynology shape | tetracolpate, elliptic in polar view | tetracolpate, elliptic in polar view |
| Palynology size | length of long equatorial axis × length of short equatorial axis: 28.05 × 16.67 μm | length of long equatorial axis × length of short equatorial axis: 27.78 × 17.50 μm |
| Palynology ornamentation | exine with an irregular reticulate ornamentation | exine with an irregular reticulate ornamentation |

**Fig. S1.** Bayesian consensus phylogram based on ITS sequences. Numbers above
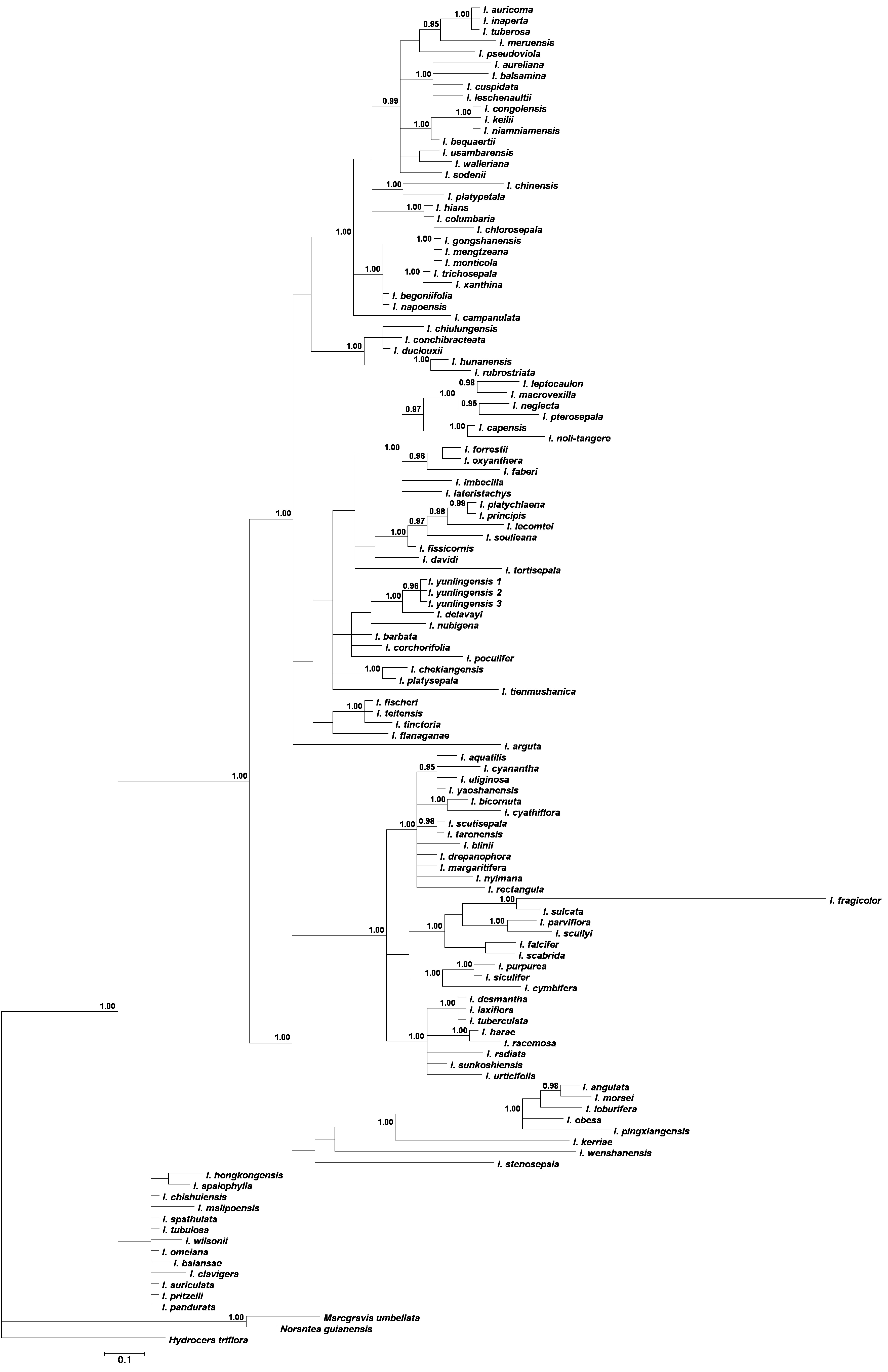
branches are Bayesian posterior probabilities (only PP values >0.95 shown).


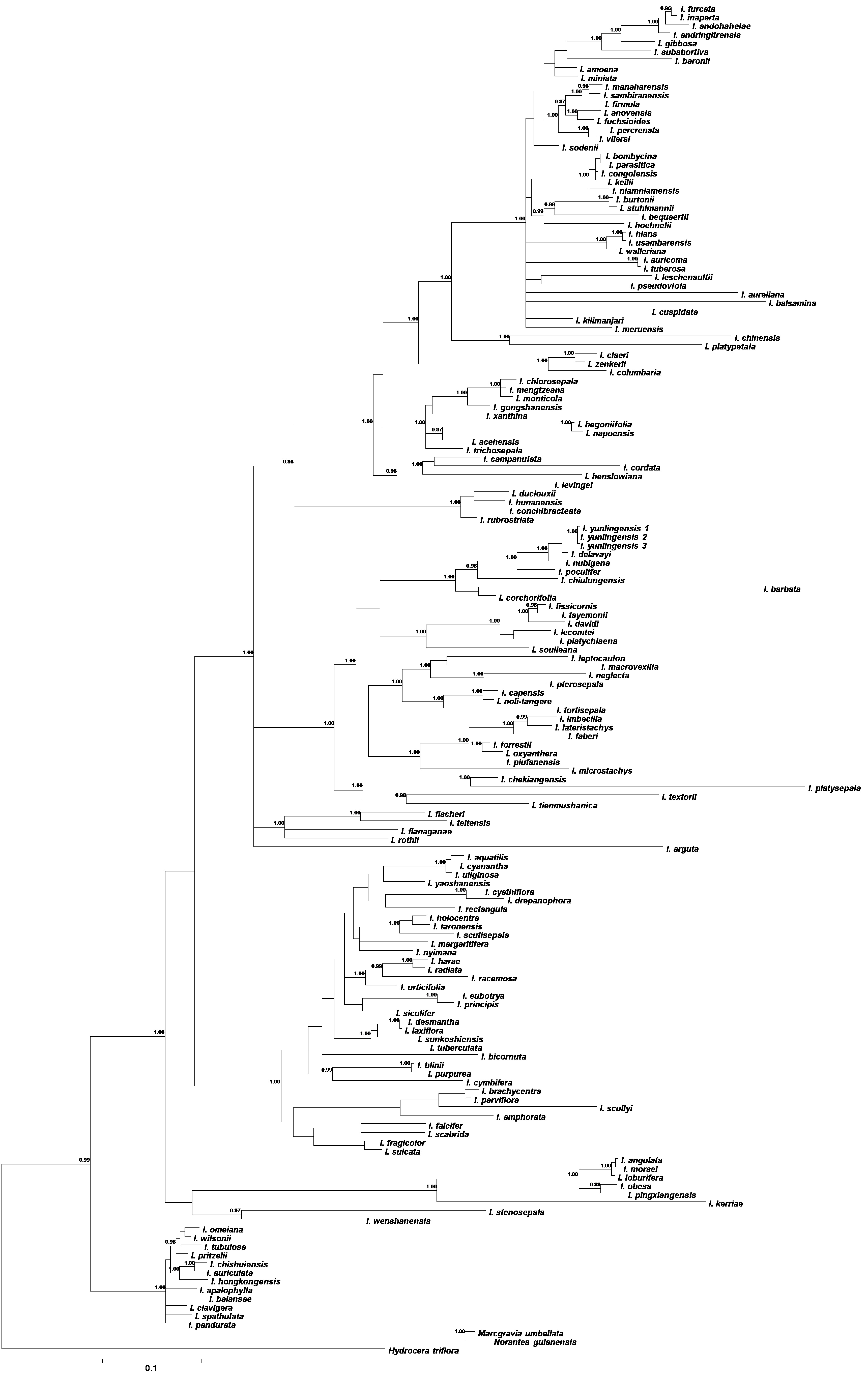
**Fig. S2.** Bayesian consensus phylogram based on *atp*B-*rbc*L sequences. Numbers above branches are Bayesian posterior probabilities (only PP values >0.95 shown).
